# Chitosan Schiff base electrospun fabrication and molecular docking assessment for nonleaching antibacterial nanocomposite production

**DOI:** 10.1007/s10570-023-05124-9

**Published:** 2023-03-15

**Authors:** Eman AboBakr Ali, Heba M. Abo-Salem, Amany A. Arafa, Ahmed A. Nada

**Affiliations:** 1 Polymer and Pigment Department, Chemical Industries Research Institute, National Research Centre (Scopus Affiliation ID 60014618), Dokki, Giza, 12622 Egypt; 2 Chemistry of Natural Compounds Department, Pharmaceutical and Drug Research Institute, National Research Centre, Dokki, Giza, 12622 Egypt; 3 Microbiology and Immunology Department, Veterinary Research Institute, National Research Centre, Dokki, Giza, 12622 Egypt; 4 Pre-treatment and Finishing of Cellulosic Fibers Department, Textile Industries Research Institute, National Research Centre, Dokki, Giza, 12622 Egypt

**Keywords:** Chitosan Schiff base nanofiber, Molecular docking, Antibacterial

## Abstract

In this work, new chitosan derivative nanofibers that exhibit antibacterial properties were successfully fabricated. The two CS Schiff base derivatives (CS-APC and CS-2APC) were prepared by incorporating 4-amino antipyrine moiety in two different ratios, followed by a reductive amination to obtain the corresponding derivatives CS-APCR and CS-2APCR. Spectral analyses were used to confirm the chemical structure. The molecular docking evaluation of CS-APC, CS-APCR, and CS was conducted on DNA topoisomerase IV, thymidylate kinase and SARS-CoV-2 main protease (3CL^pro^) active sites. CS-APCR showed a well-fitting into the three enzyme active sites with docking score values of  − 32.76,  − 35.43 and  − 30.12 kcal/mol, respectively. The nanocomposites of CS derivatives were obtained by electrospinning the blends of CS-2APC and CS-2APCR with polyvinyl pyrrolidone (PVP) at 20 kV. The morphology of the nanofibers was investigated by scanning electron microscopy (SEM). It was found that fiber diameters were significantly decreased when CS-2APC and CS-2APCR were incorporated into pure PVP to reach 206–296 nm and 146–170 nm, respectively, compared to 224–332 nm for pure PVP. The derivatives of CS and their nanofibers with PVP were found to have antibacterial activities against two strains of *Staphylococcus aureus* and *Escherichia coli.* Data revealed that CS-2APC nanofibers showed antibacterial activity to the two strains of *E. coli* less than CS-2APCR nanofibers.

## Introduction

Nowadays, generating antimicrobial materials has been of great interest in establishing the efficient treatment of bacterial infection. According to a previous report, the death of nearly two million people per year is caused by a bacterial infection (Demirci 2016). For instance, *Staphylococcus aureus* (*S. aureus*), the most infectious bacteria, is causing skin abscesses, furuncles, and cellulitis. Although most *Staph-*based infections are not serious, *S. aureus* can cause serious issues such as bloodstream infections, pneumonia, or bone and joint infections that could cause death in severe conditions (El-Hadedy and El-Nour 2012). In addition, inadequate handling of food products may lead to the rapid growth of some bacterial strains, namely, *E. coli*, *S. aureus,* and *Staphylococcus epidermidis*. Such strains produce toxins in a person’s intestine causing severe illness (Aa et al. 2016). Therefore, many efforts have been devoted to utilizing different antimicrobial agents and approaches to solve this medical issue. Recently, polymers’ activity against microorganisms has emerged and allowed polymeric matrices to be used in medical and food applications. Depending on the type of application, antimicrobial polymers can be designed as leaching, releasing the bioactive agent, or non-leaching antimicrobial polymers. The biological activity of the non-leaching polymers is acquired either from the repetition of a functional group on the polymer backbone or the attachment of a bioactive agent to the polymer chains (Siedenbiedel and Tiller 2012).

For their biocompatibility, biopolymers such as alginate, cellulose, starch, and chitin’s derivative, chitosan, have been chosen for human-related applications. Chitosan (CS) is one of the most promising biopolymer derivatives in this field due to many inherent features such as biocompatibility, biodegradability, and sustainability (Aravamudhan et al. 2014). The cationic functionality of CS is provided by repeating amino groups in its linear chains that lead to its distinguished antimicrobial activity (Ali et al. 2018). Such glucosamine repeating units of chitosan attracted more and more attention for conducting chemical modifications for numerous applications. Among all derivatives of CS, the Schiff base (SB) of CS has gained much interest. SB is derived from the reaction of the amino group of a primary amine with the carbonyl group of aldehydes or ketones. Imine functionality introduced to the polymer chains elevates the usefulness of CS as sensors, organo-catalysts, and pharmaceutical agents (Antony et al. 2019). Synthesizing chitosan Schiff bases (CSSBs) by the reaction of CS with heterocyclic aldehyde or ketone attracts more attention. For instance, Hamed et al.reported the coupling and the antimicrobial activities of three heteroaryl pyrazole aldehydes with CS (Hamed et al. 2020). Another research evaluated the antimicrobial activity of CSSBs derived from quinoline and quinazoline carbaldehydes (Haj et al. 2020). Also, the antimicrobial activity of metal complexes of CSSB prepared from the reaction of CS with dihydroxy acetophenone has been reported (Malekshah et al. 2021). To extend, biological compounds with aldehyde groups that can react with chitosan are an approach for developing new pharmaceutical polymers. In this light, antipyrine (Phenazones) and their aldehyde derivatives, a class of heterocyclic compounds, exhibit a broad range of biological activities, such as analgesic and antipyretic properties (Demirci 2016). Many researchers pay attention to this class, considering their potential as precursors for substrates with numerous activities. More precisely, 4-amino antipyrine derivatives were utilized as effective antitumor, anti-inflammatory and antiviral drugs.

On the other hand, the fabrication of easily processed antimicrobial substrates with controlled release has remained challenging for a long time. With emerging technological advances in biomedicine, electrospun nanofibers are gaining momentum. It is the most widely used method to fabricate nanofibers for drug delivery, regenerative medicine, wound healing, and tissue engineering (Aravamudhan et al. 2014; Wei et al. 2020; Nada et al. 2020). Electrospinning involves the injection of a charged polymer solution/mixture towards a collector with an opposite charge using a metallic needle (spinneret). In this case, a Taylor cone is generated at the needle tip to form a spiral jet of polymer solution collected as solid nanofibers on the oppositely charged collector (Nada et al. 2018). The greatness of this technique arises from the ability to adopt different strategies to enhance the properties of the fabricated matrix. The coalescence of this technology with natural and synthetic biopolymers is desired in the medical section. Polyvinyl pyrrolidone (PVP) is one of the most widespread polymers that matches the needs of biomedical applications (Kurakula and Koteswara Rao 2020). It possesses biocompatibility, lack of irritation to biological systems, no or low toxicity, and water solubility. In addition, PVP amphiphilic nature has expanded PVP usage in many applications. PVP has a polar lactam group in pyrrolidone that provides hydrophilicity, and a non-polar methylene moiety provides lipophilicity. (Koczkur et al. 2015). Because of its spinnability and fiber extraction, PVP and its blends were widely used to fabricate nanofibers (Kurakula and Koteswara Rao 2020; Utkarsh et al. 2020; Zahran et al. 2020).

In this work, nanofibers with antibacterial properties were successfully fabricated using CS derivatives and PVP. First, CSSB was prepared by incorporating 4-amino antipyrine moiety followed by a reductive amination reaction. The molecular docking studies of CS derivatives were carried out. The morphology of CS/PVP composite electrospun nanofibers with different ratios of CS derivatives was investigated. The antibacterial activities of CS derivatives and CS derivatives/PVP nanofibers were evaluated.

## Experimental

### Materials

Chitosan (CS) low molecular weight (50,000–190,000 Da, 75–85% deacetylated) (Iceland), polyvinyl pyrrolidone (PVP) average mol wt 360,000 (China) and 4-Antipyrinecarboxaldehyde (APCA) (Eschenstrasse, Germany) were purchased from Sigma-Aldrich. Sodium Borohydride (NaBH_4_) was a product of Alfa (Ward Hill, USA). Acetic acid (glacial), ethanol and acetone were provided by a local company (Pio Chem., Cairo, Egypt).

### Preparation of chitosan-4-antipyrine Schiff base (CS-APC)

The experiment was carried out as chitosan reacted with APCA in two molar ratios; 1:1 and 1:2, respectively. In a typical procedure, 0.5 g of CS was suspended in 15 mL of ethanol solution of APCA and stirred for two hours. After that, 1 mL of glacial acetic acid was added, followed by the addition of distilled water to form a 2% acetic acid solution. The reaction proceeded for 24 h at 50 °C. After cooling, the mixture was poured into a Teflon dish and air-dried for 24 h. The product (CS-APC or CS-2APC) was washed with ethanol to remove the excess APCA and the residual acetic acid.

### Reductive amination of chitosan-4-antipyrine Schiff base (CS-APCR)

**Scheme 1:**
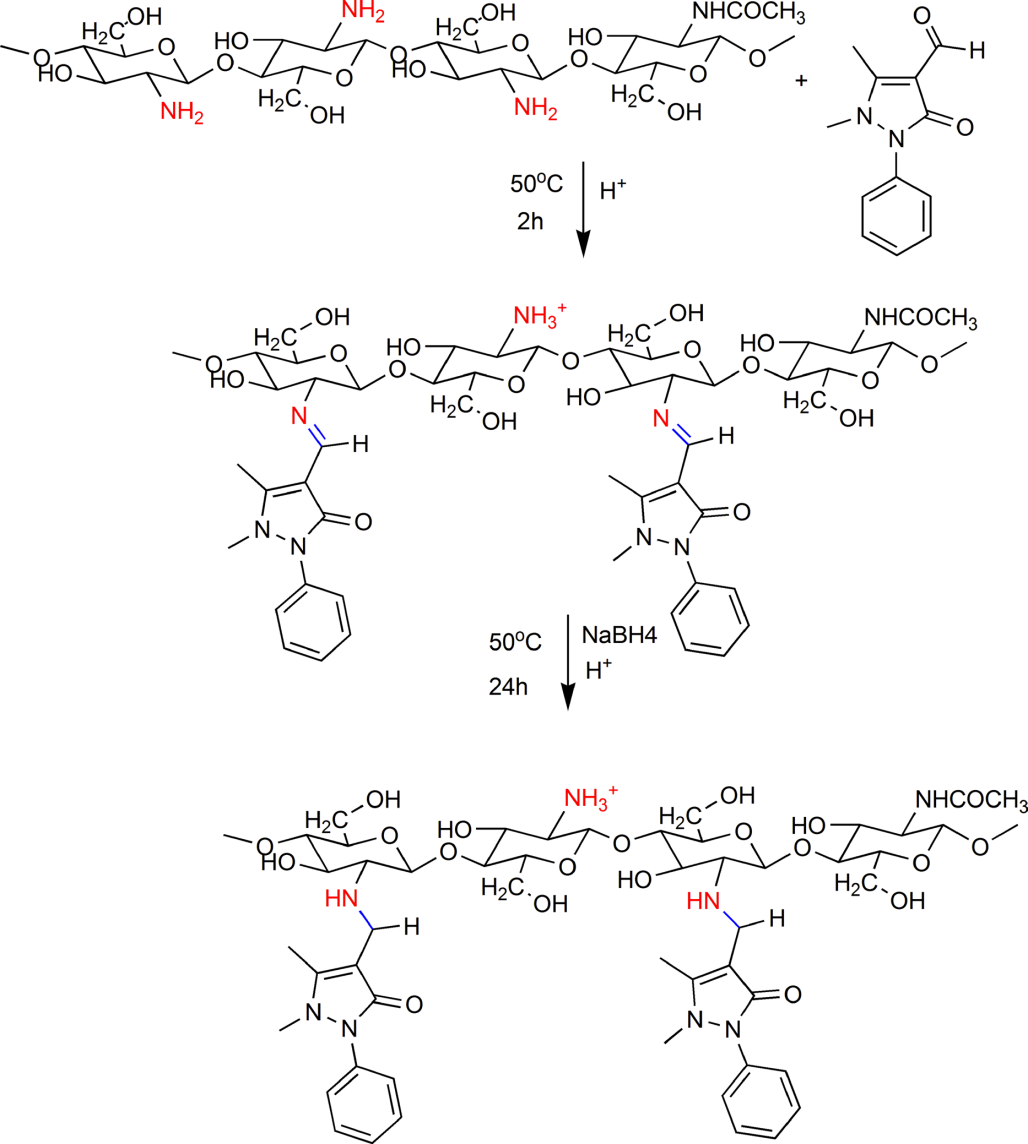
CS- APCA Schiff-base formation and the reductive amination

Previously prepared CS-APC (0.5 g) was dissolved in 25 mL of 2% acetic acid solution. Then, 0.3 g of NaBH_4_ was added slowly, and the reaction was kept for 4 h with continuous stirring (Scheme 1). The product was collected after precipitation by acetone and dried for 24 h at room temperature.

### Electrospinning

The electrospinning solutions were prepared by mixing different concentrations of (CS-2APC) and (CS-2APCR), the highly substituted CS derivatives, with PVP. Typically, 3% w/v (CS-2APC) and (CS-2APCR) in distilled water were mixed with different volumes of 10% w/v PVP solution in ethanol to end up with 3%, 6%, and 9% of the CS derivatives to PVP. The mixed solutions of Schiff base chitosan derivative denoted as; PVP-3%CS-2APC, PVP-6%CS-2APC, and PVP-9%CS-2APC were applied for electrospinning by the home-made electrospinning apparatus at 20 kV, 15 cm, and with a flow rate of 0.7–0.8 mL/h. Two concentrations of reduced chitosan derivative (CS-2APCR) were tested for electrospinning and denoted as PVP-3% CS-2APCR and PVP-6% CS-2APCR.

### Characterization

#### *Proton nuclear magnetic resonance spectroscopy (*^*1*^*H-NMR)*

^1^H-NMR of the polymers was recorded using a JEOL-ECA-50 NMR instrument 500 MHz, USA. Dimethyl sulfoxide (DMSO) and trifluoroacetic acid were taken as chemical shift references.

#### Fourier transform infrared spectroscopy (FT-IR)

FT-IR spectra were measured by FT-IR instrument model 460 plus Jasco, Japan, at Micro Analytical Center, Cairo University. The measurement range was 400–4000 cm^−1^ with a 4 cm^−1^ resolution.

#### Determination of degree of substitution

Using the Kjeldahl method (Ding et al. 2014), nitrogen content (N%) was determined for CS and its derivatives. Then, the degree of substitution (DS) was calculated using the following equation:

1 $$DS = 1 - \left( {\frac{{W_{b} }}{{W_{a} }}\frac{{N_{b} }}{{N_{a} }}} \right)$$

where W_a_ and W_b_ are the molar quantity of glucosamine unit and the modified chitosan substituted, respectively. N_a_ is the nitrogen content of untreated chitosan and N_b_ is the nitrogen content of the modified chitosan.

#### Scanning electron microscopy (SEM)

The electrospun fiber morphology was assessed using scanning electron microscopy (Quanta 250 FEG (Field emission Gun), USA, located at the National Research Centre (NRC). Electrospun mats, deposited on an aluminum sheet, were fixed to the sample holder and coated with a layer of gold in a vacuum using a sputter coater (S150 A Edwards-England). Gold-coated mats were placed in the microscope chamber. Features of sample morphology were obtained in 5–10 kV. Fiber diameters were estimated from the SEM micrographs using ImageJ software (ImageJ 1.52 h, USA).

### Molecular modeling

#### Target preparation

##### Anti-bacterial target preparation

The crystallographic structure of DNA topoisomerase IV subunit B and thymidylate kinase (TMPK) were retrieved from the protein data bank at using 4HZ5 and 4QGG codes (Tari et al. 2013; Kawatkar et al. 2014), respectively. Water molecules were removed, hydrogen atoms and partial charges were added, then a Gaussian contact surface around the binding sites was drawn using the MOE program (Molecular Operating Environment, Version 2019.01, Chemical Computing Group Inc., Montreal, Canada).

##### SARS-CoV-2 protease preparation

The crystallographic structures of SARS-CoV-2 main protease (3CL^pro^) were retrieved from the protein data bank at using 6LU7codes and loaded onto the software. The protein was prepared using the MOE QuickPrep tool module, and the active site was identified.

##### Ligand preparation

The 2D structures of trimers CS-APC, CS-APCR, and CS (3mers) were generated using ChemDraw Ultra 12.0, then converted into 3D. The structure was minimized using the MMFF94x force field (eps = r, Cutoff until the RMSD gradient of 0.01 kcal mol^−1^ Å^−1^ was adopted). The protonation state of each compound was assigned based on the neutral pH. The compounds were imported into the MOE database for docking.

#### Docking procedures

Docking simulations were performed by the MOE program using the induced-fit (flexible) protocol. The Triangle Matcher placement method was used to place ligand conformations in the active site, and then they were ranked using the London ∆G scoring function1 and refined with a force field. To validate our docking protocol, the co-crystallized ligands of the target proteins were re-docked into the enzyme active sites. The applied protocol is acceptable if the re-docked ligands show a low RMSD of less than 2 Å^−1^. Docking of the CS derivatives into the binding sites of selected enzymes (DNA topoisomerase IV subunit B, thymidylate kinase, and main SARS-CoV-2 protease (3CLpro)) was performed. The docking simulation created 30 poses for each compound, sorted by the lowest binding energy.

### Antimicrobial study

#### Microorganisms and culture conditions

Bacterial strains used in this study are from American Type Culture Collection (ATCC) strains and local strains that isolated and identified from normal buffalo milk samples. The bacterial strains isolated from animal origin were isolated and identified according to (Quinn et al. 2011). Briefly, under complete aseptic condition, 1 mL of milk sample was added to 10 mL peptone water and vigorously shaken, then incubated for 8 to 12 h at 37 °C. Ten microliters of each sample were inoculated on the mannitol salt agar plate, and the MacConkey agar plate then incubated at 37 °C for 18 to 24 h. The colonies isolated from the mannitol salt agar plate were identified as *staphylococci* based on colony morphology, Gram staining, catalase test, oxidase test, and oxidative fermentative test. After the confirmation of the genus *Staphylococcus*, the enzyme coagulase was characterized using both slide and tube methods. Coagulase-negative *Staphylococcus* isolate was subjected to identification to the species level using the API STAPH commercial identification system (API STAPH bioMerieux® SA 69,280 Marcy- 1’Etoile/France) (Thorberg et al. 2006; Aa et al. 2016) which were identified as *S. aureus*. While colonies isolated from the MacConkey agar plate were identified as *E. coli* based on colony morphology, Gram staining, oxidase test, the indole, methyl red, Voges-Proskauer and Simmons citrate (IMVic) test as putatively *E. coli*. The strains were subsequently characterized by the biochemical API 20E test system (Biomerieux) according to the manufacturer’s instructions and identified serologically as *E. coli* sero type O27. The identified strains were sub-cultured. The colonies were transferred to Todd Hewitt broth (Becton Dickinson Diagnostic Systems, Sparks, MD). After that they were cultured at 37 °C for 18 h and stored in 20% glycerol solution at 80 °C for further use.

#### Antibacterial test

The antibacterial activity of the samples was tested against the American Type Culture Collection (*E. coli* ATCC 25,922 and *S. aureus* ATCC 33,591) and two isolates from animal origin, milk samples, (*E. coli* sero type O27 as Gram-negative bacteria and *S. aureus* as Gram-positive bacteria). The antibacterial test was performed quantitatively using the standard test method according to the AATCC test method 100–1999 for bacterial counting (Chun et al. 2009). All tests were conducted by using the standard plate count technique. All samples were prepared at a standard weight. Each microorganism used in this experiment was individually inoculated into tubes containing 5 mL BHI (brain heart infusion broth) sterile suspension. The suspension was adjusted to 0.5 McFarland standards to match the turbidity of 1.5 × 10^–8^ mL^−1^ colony forming unit (CFU) (Chun et al. 2009). All samples were kept at a controlled temperature of 37 °C. After incubation, it was transferred into 100 mL of nutrient broth (1:500) and mixed vigorously for 1 min. A tenfold dilution with 0.9% (w/v) normal saline solution was prepared, and spread at varying dilutions onto plates containing MacConkey agar for *E. coli* and the mannitol salt agar for *S. aureus*. Incubation to all plates was done at 37 °C for 24 h. All experiments were performed in triplicate. The antibacterial activity is expressed in the reduction percentage of the organisms after making contact with the test specimen compared to the number of the organism cells surviving after making contact with the control. All results were expressed according to the following equation:

2 $$Reduction \left( \% \right) = \left( {\frac{B - A}{A}} \right)*100$$

where B is the number of microorganisms present on untreated samples, and A is the number of microorganisms present on treated samples.

#### Statistical analysis

All measurements are the average of three replicates; the results were processed by analysis of variance. The significance was determined by the least significant difference (LSD) levels of 1% and 5%.

## Results and discussion

Chitosan Schiff bases are consistently formed by the condensation of chitosan's amino groups with the carbonyl groups of either aldehydes or ketones through the withdrawal of water molecules. Through extensive experimental and theoretical investigation, it was found that the reaction mechanism depends more or less on the pH, solvent, and reactants properties (Erdtman et al. 2011; Setamdideh and Sepehraddin 2014; Antony et al. 2019).

**Fig. 1:**
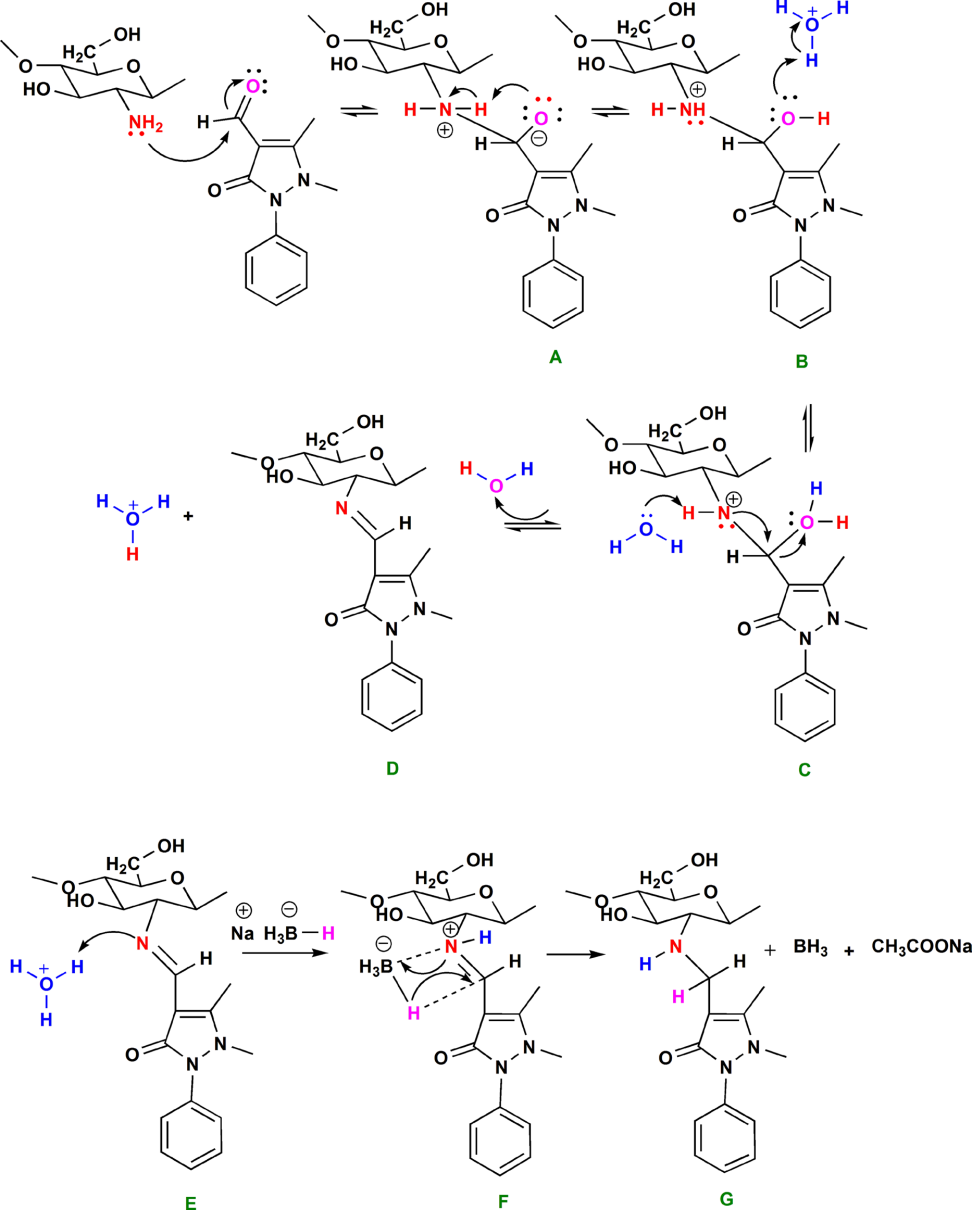
The proposed mechanism of CS-APCA Schiff-base formation and the reductive amination

Figure 1 illustrates the prospective reaction mechanism of APCA with Chitosan. The reaction procedure started with chitosan suspended in ethanol to produce a zwitterionic carbinolamine intermediate (**A)** as a result of the nucleophilic attack of the chitosan unprotonated amino group to the aldehyde carbonyl group. In the second step, upon the addition of water and acetic acid, protonation of the OH group leads to the breaking the C–OH bond. In an acidic medium, the dehydration of (**B)** is accelerated (Erdtman et al. 2011). In the intermediate compound **C**, the water molecule has dual roles; it acts as a hydrogen donor at the adjacent OH group and a hydrogen acceptor at the NH group that facilitates the formation of the Schiff base derivative of chitosan (**D)**. In this stage, a Schiff base derivative of chitosan (compound **D)** is formed and purified for further investigation. The corresponding 2^o^amine was obtained by subjecting** D** to a metal-reducing agent, NaBH_4_, in an acidic medium to protonate the nitrogen of the imine. The imine intermediate (**E)** facilitates the hydrogen addition to the nearest adjacent carbon to form the 2^o^amine.

### ***FT-IR and ***^***1***^***H-NMR analysis***

**Fig. 2:**
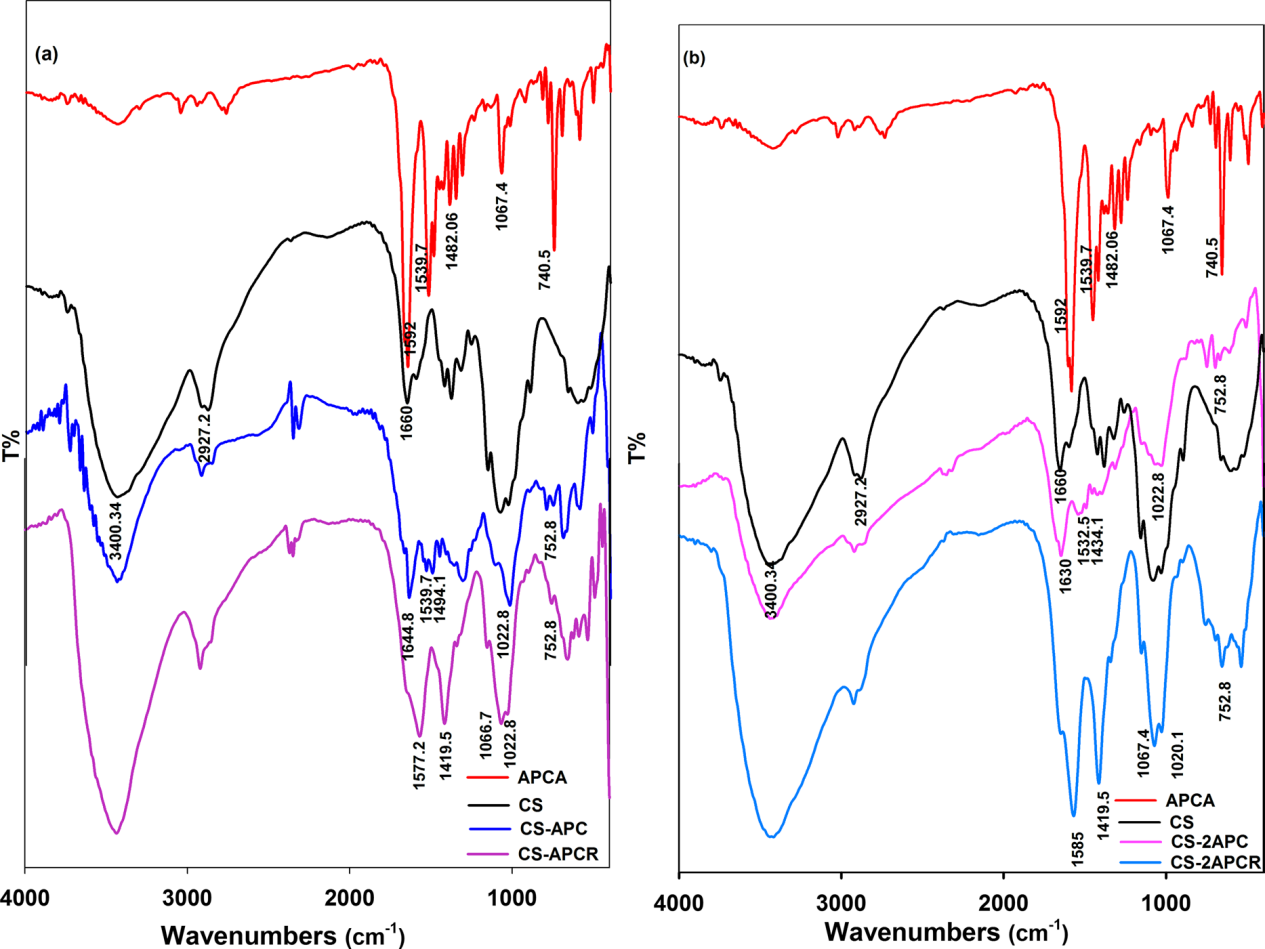
FT-IR of CS and its drevatives: **a** CS/APCA mole ratio (1:1), and **b** CS/APCA mole ratio (1:2)

The FT-IR spectrum of CS (Fig. 2a, b) shows the characteristic, broadband at 3400 cm^−1^ that is assigned to the inter- and intra-molecular interaction between the OH and NH_2_ groups. In addition, there are two bands at 1650.01 cm^−1^ assigned to the amide I (C=O stretching) and the amide II (N–H stretching) (Ali et al. 2020). Moreover, two bands were observed at 2920.66 and 2365.22 cm^−1^ corresponding to the symmetrical and asymmetrical stretching of the methylene groups in residual chitin molecules. FT-IR spectrum of CS derivative confirms the production of chitosan Schiff base through observing a decrease in the intensity of N–H peak at the region 3000–3500 cm^−1^ (Silverstein et al. 2005). This decrease is directly proportional to the increase of the molar ratio of APC. Figure 2 shows the presence of the imine bond (N=C) at 1645 cm-1 for CS-APC and CS-2APC a long with the absence of aldehyde peak of APC. In addition, there is a stretching vibration band assigned to the C–C bond of the APC aromatic ring at 1484 cm^–1^. The aromatic bending in the plane of the C–H bond stands at 1057 cm^–1^ (Hamed et al. 2020). The C=N stretching peak in the range of 1595–1547 cm^−1^ is assigned to the pyrazole moiety of APC. After the reductive amination step, the N–H peak intensity is enlarged, and C-H characteristic peak 1390 cm^−1^ appeared in CS-APCR and CS-2APCR spectrum (Silverstein et al. 2005; Nikmawahda et al. 2015).

**Fig. 3:**
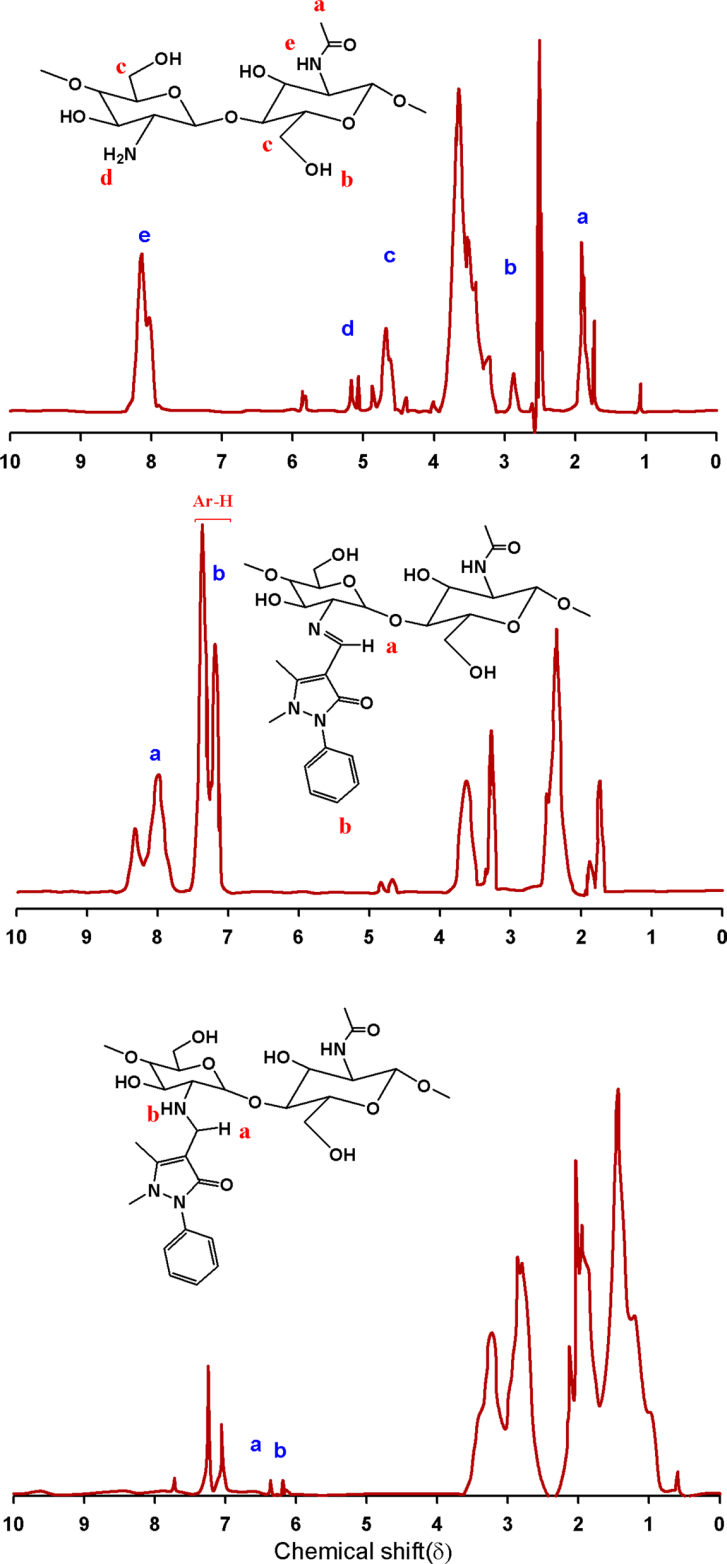
1H-NMR of **a** CS, **b** CS-2APC and CS-2APCR

The ^1^H-NMR spectra of CS, CS-2APC, and CS-2APCR are shown in Fig. 3. These samples were dissolved in a mixed solvent of trifluoroacetic acid and DMSO (500 MHz, TFA/DMSO). The ^1^H-NMR spectra track the change of CS amino group peaks. Generally, the shift of NH_2_ protons depends on concentration, temperature, and solvent (Pavia et al. 2001). Using trifluoroacetic acid as a protonating agent as well as a solvent grants the classification of the primary and secondary amines in the chemical shift region 6 to 8.5δ. The signals were in agreement with data reported elsewhere (Marin et al. 2013, 2015; Triana-Guzmán et al. 2018). The ^1^H-NMR spectrum of CS (Fig. 3a) showed broad signals at δ 8.14 for NH_2_ groups, 5.16 for Hb, 4.68 for Hd, and 1.73 for the proton of the methyl group (NHCOCH_3_), in addition to the other characteristic signals for chitosan protons. The Schiff base CS-2APC ^1^H-NMR spectrum (Fig. 3b) illustrated new broad signals at δ 8.01 assigned to the anil proton (CH=N), and 7.38 − 7.20 assigned to the aromatic protons of antipyrine moiety. In addition, the data revealed multiple signals at δ 4.83–2.35 that are assigned to the methylene groups (CH_2_OH), OH groups, and the two methyl groups attached to the pyrazole ring. CS-2APCR ^1^H-NMR spectrum illustrated the lake of anil proton signal and revealed new signals at chemical shift 6.50 and 6.35 for NH protons and CH protons.

The degree of substitution (DS) calculated based on the nitrogen content (%) analysis data is attested in many literature papers (Ding et al. 2014; Mohamed et al. 2020). Nitrogen content was obtained by using the Kjeldahl method reporting 4.93% for CS, 3.96 for CS-APC, and 3.2% for CS-2APC. Accordingly, DS was found to reach 0.01 and 0.02 for CS-APC and CS-2APC, respectively. Further electrospinning and antibacterial investigation were carried out using the highly substituted product, CS-2APC.

### Fiber formation and morphology

**Fig. 4:**
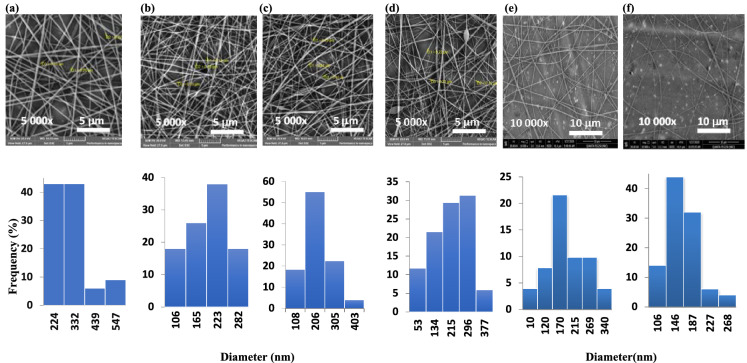
SEM images of **a** PVP, **b** PVP-3%CS-2APC, **c**PVP-6%CS-2APC, **d** PVP-9%CS-2APC, **e** PVP-3%CS-2APCR and **f** PVP-6%CS-2APCR

Polyvinyl pyrrolidone (PVP), high molecular weight, was used to enhance both the electro-spinnability of CS-2APC and CS-2APCR and to provide excellent antimicrobial activities for the final nanocomposite. The nanofiber’s SEM images (Fig. 4) illustrate the effect of CS-2APC and CS-2APCR concentration on the nanofiber morphology. Data revealed that loading 3 to 6% CS-2APC to PVP solutions yielded continuous bead-free and relatively homogenous distributed electrospun fibers. Beyond this range, 9% of CS-2APC lead to some beads and spindle-like fibrous in the matrix and randomly distributed nanofibers with a diameter of 50–374 ± 3 nm. However, the addition of more than 6% of CS-2APCR forms noncontinuous fibers with more beads. Thus, only 3 and 6% were considered for CS-2APCR. The nanofibers of PVP- CS-2APCR exhibited uniform fibers where nanofiber diameters were 170 ± 1 and 150 ± 6 nm for 3 and 6% of CS-2APCR, respectively. In agreement with other reports (Nada et al. 2020; Liu et al. 2021), introducing chitosan derivatives in a spinning solution reduces the nanofiber diameter. This behavior is attributed to the ability of CS to increase the charge concentration on the jet, which leads to more repulsion on the surface resulting in a decrease in the fiber diameter.

### Docking studies

**Fig. 5:**
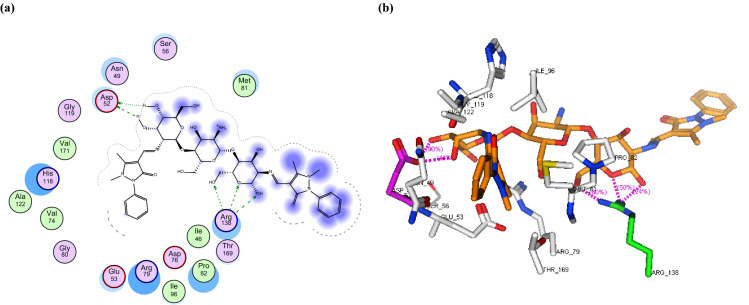
**a** 2D binding interaction and **b** 3D (brown, stick) binding mode of CS-APC within the active site of topoisomerase IV subunit B (PDB ID: 4HZ5

**Fig. 6:**
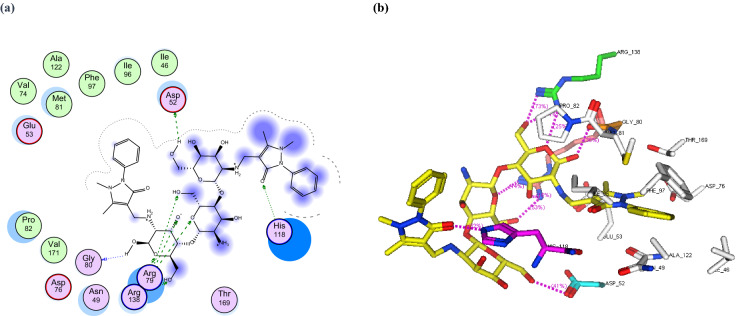
**a** 2D binding interaction and **b** 3D (yellow, stick) binding mode of CS-APCR within the active site of topoisomerase IV subunit B (PDB ID: 4HZ5)

**Fig. 7:**
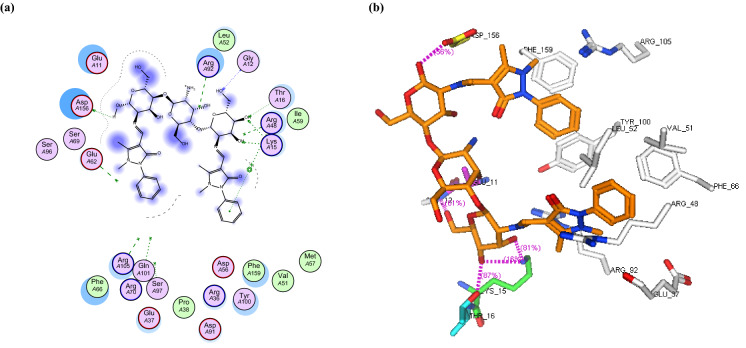
**a** 2D binding interaction and **b** 3D (brown, stick) binding mode of CS-APC within the active site of thymidylate kinase (PDB ID: 4QGG)

**Fig. 8:**
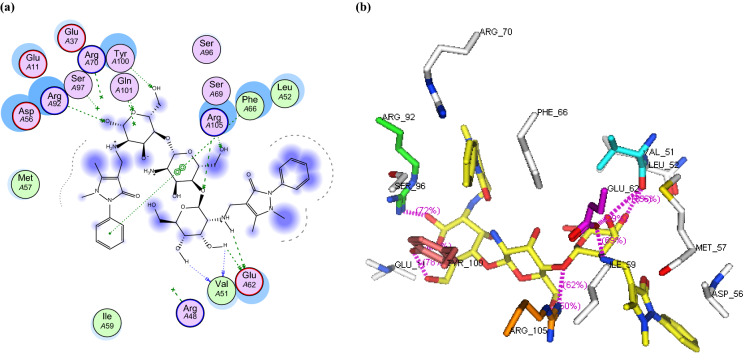
**a** 2D binding interaction and **b** 3D (yellow, stick) binding mode of CS-APCR within the active site of thymidylate kinase (PDB ID: 4QGG)

**Fig. 9:**
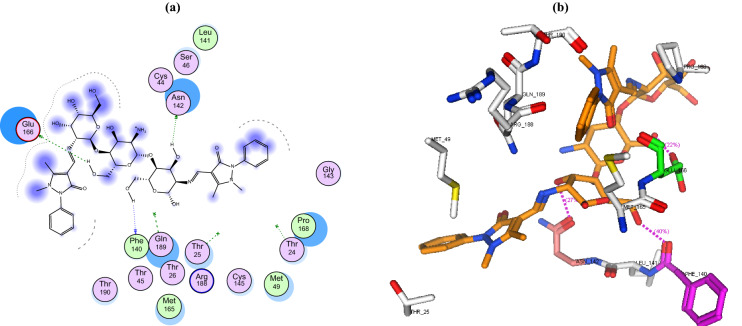
**a** 2D binding interaction and **b** 3D (brown, stick) binding mode of CS-APC within the active site of CoV-2 main protease (PDB ID: 6LU7)

**Fig. 10:**
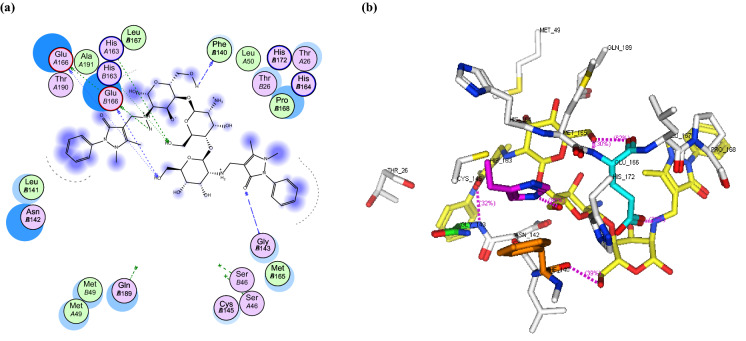
**a** 2D binding interaction and **b** 3D (yellow, stick) binding mode of CS-APCR within the active site of SARS-CoV-2 main protease (PDB ID: 6LU7)

**Fig. 11:**
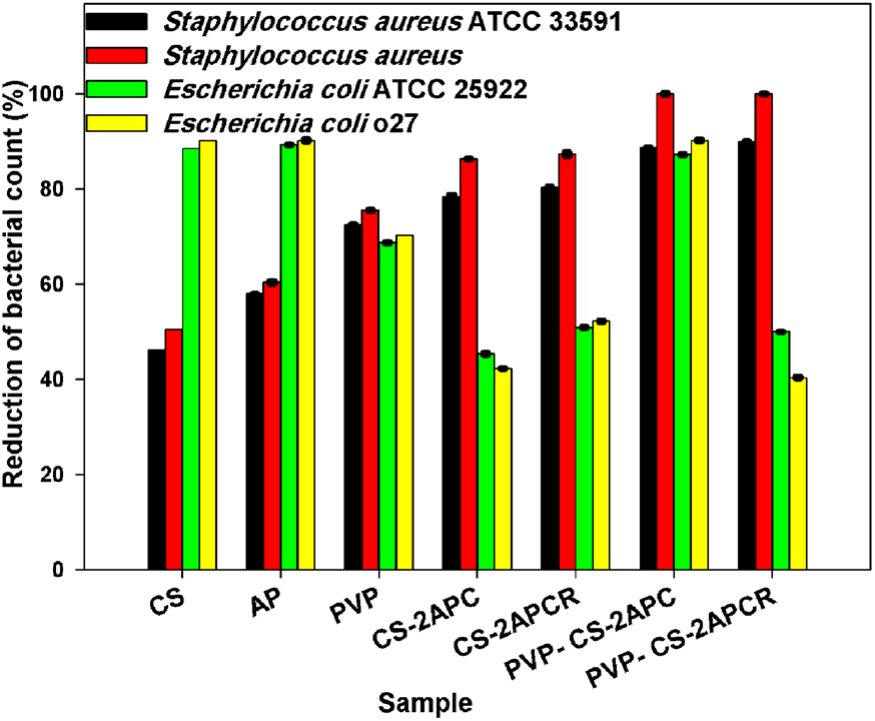
The reduction percentage of the organisms by the samples

With the increasing demand for developing new drugs, drug designing by computer-aided drug design has become an essential tool. It offers various approaches to comprehending chemical systems, delivering knowledge that is difficult to gain in laboratory research and at a far lesser cost and effort than experiments (Tomar et al. 2019). In the current study, we employed the molecular docking technique to investigate the binding mode of newly synthesized chitosan derivatives with assorted biological targets, comparing the results to chitosan. The docking findings demonstrate that the novel chitosan derivatives have higher antimicrobial activities than chitosan (Table 1, Figs. 5, 6, 7, 8, 9, 10, 11).

**Table 1:** Docking results of CS-APC, CS-APCR and CS towards the antibacterial targets DNA topoisomerase IV, thymidylate kinase

Comp. No.	Moieties from the compound	Amino acid residues	Type of interaction Distance Å	Score Kcal/mol
*DNA topoisomerase IV subunit B (ID: 4HZ5)*				
Native ligand	Pyridine ring	Arg79	Pi-cation	− 15.22
				RMSD: 0.81
CS-APC	OH	Asp52	H-don, 1.66, 1.48	− 28.53
	OH	Arg138	H-acc, 2.49, 2.64 and 2.82	
CS-APCR	OH	Asp52	H-don, 1.95 and 1.29	− 32.76
	C=O of pyrazole ring	His118	H-acc, 2.51	
	OH, O	Arg79	H-acc, 3.23, 2.94 and 2.56	
	OH	Gly80	H-don 1.64	
	OH, O	Arg138	H-acc 3.15, 2.63 and 2.88	
CS	OH	Asp52	H-don, 3.74 and 3.74	− 11.21
	OH	Asp76	H-don, 1.26	
*Thymidylate kinase (ID: 4QGG)*				
Native ligand	NH, CO	Gln101	H-don, 1.75 and 2.88	− 28.34 RMSD: 1.65
	O, CO	Arg 48	H-acc 22.6% 2.91, 2.81	
	CO	Arg70	H-acc 63.2% 2.86	
	CO	Ser97	H-acc 70.8% 2.73	
CS-APC	CO	Lys15	H-acc, 3.15 and 2.77	− 29.69
	OH	Gly12	H-acc, 2.86	
	H	Asp156	H-don, 1.37	
	O	Thr16	H-don, 2.65 and 2.65	
	Phenyl ring	Lys15	*Π*-Cation	
CS-APCR	OH	Val51	H-don, 1.83 and 3.57	− 35.43
	NH and OH	Glu62	H-don, 1.41 and 1.26	
	O and OH	Arg105	H-acc, 2.59 and 2.89	
	OH	Tyr100	H-acc, 2.46, 3.28 and 2.46	
	OH	Arg92	H-acc, 2.66	
	Phenyl ring	Phe66	*π* − *π*	
CS	OH	Glu37	H-don, 1.80	− 13.85
	NH	Glu62	H-don, 1.18	
	OH	Arg105	H-acc, 2.49	
	NH2	Tyr100	*π*-Cation	
	OH	Pro168	H-don, 1.71

#### Anti-bacterial targets

Antibiotic resistance (AMR) is among the public health challenges of the twenty-first century because it is a threat to the prevention and treatment of a growing number of microbial, parasites, viruses, and fungi diseases that are no longer treatable to antibiotics (Prestinaci et al. 2015). Accordingly, developing agents with distinct modes of action has been of great interest. Given the reported observation that compounds interacting with multiple enzyme active sites can diminish microbial resistance, the possibility for inhibiting antibiotic resistance is elevated (Silver 2007).

In this study, the binding mode of the newly synthesized chitosan derivatives CS-APC and CS-APCR were investigated towards two bacterial enzymes topoisomerase IV subunit B (PDB ID: 4HZ5) and thymidylate kinase (PDB ID: 4QGG) by the MOE software in comparison to chitosan (CS). In the case of topoisomerase IV, the result revealed that CS-APCR exhibited a better docking score (−32.76 kcal/mol) than CS-APC, CS, and the native ligand  − 28.53,  − 11.21 and  − 15.22 kcal/mol, respectively. Also, CS-APCR showed** a** better binding interaction with the enzyme active site via the formation of three H-bonds acceptor with the essential amino acid residues in the active pocket Arg79 similar to native ligands beside three H-bonds Donner with Asp52 and Gly80 and four H-bonds acceptor with His118 and Arg138 (Table 1, Fig. 6). Likewise, in case of thymidylate kinase (PDB ID: 4QGG), CS-APCR revealed a powerful docking score  − 35.43 kcal/mol than CS-APC, CS and the native ligand  − 29.69,  − 28.34 and  − 13.85 kcal/mol, respectively (Table 1). Furthermore, CS-APCR exhibited superior binding affinity with the enzyme active site through the generation of ten hydrogen bonds; four H-bonds Donner with amino acid residues Val51 and Glu62 and six H-bond acceptors with Arg105, Tyr100 and Arg92, in addition, *π *− *π* interaction between phenyl ring of antipyrine moiety and Phe66 residue (Fig. 8). Although the fact that CS-APC had a lower docking score than CS-APCR, its performance is better than CS and the native ligand with the two enzymes. Also, CS-APC demonstrated stronger binding affinity within the active sites (Table 1, Figs. 5, 7).

##### COVID-19 target

SARS-CoV-2 main protease, a chymotrypsin-like cysteine protease (3CL^pro^) which plays an vital role in mediating the replication and transcription of viruses, is considered an attractive target for the discovery of antiviral agents (Calligari et al. 2020). From the Protein Data Bank, the crystal structure of the 3CL main protease in complex with a peptide-like inhibitor N3 (PDB ID: 6LU7) (Jin et al. 2020) was obtained and prepared for the docking process utilizing MOE software. The result indicated that CS-APC and CS-APCR had similar binding energies of  − 30.82 and  − 30.12 kcal/mol, respectively, that is better than CS (**−**20.79 kcal/mol) (Table 2). However, CS-APCR had the best binding affinity with the enzyme active site, forming six hydrogen bonds with the key amino acid Glu166 besides six hydrogen bonds with Phe140, Gly143 and His163 residues (Table 2, Fig. 10). In addition, CS-APC was able to stabilize into the active pocket via formation of three hydrogen bonds as a Donner with amino acid residues Glu166, Phe140 and Asn142 (Table 2, Fig. 9).

**Table 2:** Docking results of CS-APC, CS-APCR and CS on SARS-CoV-2 main protease (PDB ID: 6LU7)

Comp. No.	Moieties from the compound	Amino acid residues	Type of interaction Distance Å	Score Kcal/mol
CS-APC	OH	Phe140	H-don, 1.71	− 30.82
	OH	Glu166	H-don 1.30	
	OH	Asn142	H-don 26.9% 1.96	
CS-APCR	OH	Phe140	H-don, 1.87 and 1.87	− 30.21
	OH and NH	Glu166	H-don, 1.25, 1.25, 2.80, 2.80 and H-acc, 2.62 and 2.62	
	CO of pyrazole ring	Gly143	H-acc, 2.67 and 2.67	
	OH	His163	H-acc 2.75 and 2.75	
	OH	Phe140	H-don, 1.87 and 1.87	
CS	OH	Asn142	H-don, 1.45	− 20.79
	OH, NH	Glu166	H-don, 1.25, 1.51, 3.11 and H-acc, 2.80	
	OH	Pro168	H-don, 1.71	

### Antimicrobial assessments

4-amino antipyrine derivatives are considered an outstanding drug in pharmacology. Introducing such moiety to CS polymer chains as a side functional group is likely providing a non-leaching antibacterial polymer. In this assessment, the antibacterial activities of CS-2APC, CS-2APCR, and their electrospun nanofibers against two types of bacteria, Gram-positive and Gram-negative bacteria, were investigated. This study was conducted against the American Type Culture Collection (*E. coli* ATCC 25,922 and *S. aureus* ATCC 33,591) and two strains isolated from milk samples (*E. coli* sero type O27 as Gram-negative bacteria and *S. aureus* as Gram-positive bacteria). CS, a cationic biopolymer, shows antimicrobial activity owing to its ability to bind and destroy the negatively charged components in cell membranes (Sahariah and Másson 2017). This manner is the dominant mode of action of chitosan towards Gram-negative bacteria. However, there is another mode of action where chitosan is assumed to target the microorganism intracellular. According to several studies, chitosan can penetrate the multilayered (murine cross-linked) bacterial cell wall and the cytoplasmic membrane and interact with the DNA. Consequently, CS prevents DNA transcription and interrupts protein and mRNA synthesis. (Fei Liu et al. 2001). This mode of action is likely accepted for low molecular weight chitosan (Sahariah and Másson 2017).

During this investigation, the substitution of the CS amino group by Schiff base derivative might be the reason for decreasing the CS antibacterial activity against *E. coli* (Fig. 11). This behavior was previously reported by (Yin et al. 2012; Antony et al. 2019). This research group demonstrated that Schiff bases (CSSBs) containing electron-withdrawing substituents are better antimicrobial agents against *E. coli* and *S. aureus* than the (CSSBs) containing electron-donating substituents.

In our study, a slight increase in the antibacterial activity was observed for CS-2APCR compared to CS-2APC. However, the best antibacterial activity of the nanofibers is more likely to be attributed to their higher surface area-to-volume ratio.

It can be concluded from Fig. 11 that the attachment of APC to CS has enhanced CS antibacterial activity against *S. aureus* ATCC 33,591 by 69% and 74% for CS-2APC and CS-2APCR, respectively. Even with the highly cross-linked thick cell wall of *S. aureus*, it seems to have a vital role played by APC in *S. aureus* inhibition. In the case of Gram-negative bacteria, it has been reported (Vinsova and Vavrikova 2011; Sahariah and Másson 2017) that blocking the outer cell wall is an effective antibacterial mode of action. Therefore, the formed layer by CS derivatives prevents nutrient flow and augments an internal osmotic pressure. The deficiency of nutrient flows into the cell, and the hindrance of metabolize to exiting from the cell is most likely the main reason for cell death.

## Conclusion

Novel CS derivatives/PVP nanofibers that exhibit antibacterial properties were successfully fabricated. The two CS derivatives were prepared by introducing 4-amino antipyrine moiety chemically to CS, followed by reductive amination to obtain the stable corresponding amine derivatives. Chemical structures of the obtained CS derivatives were confirmed via spectral analysis. Docking studies of CS-APC, CS-APCR, and CS were conducted on DNA topoisomerase IV, thymidylate kinase, and SARS-CoV-2 main protease (3CL^pro^) active sites. CS-APCR displayed a well-fitting into the three enzyme active sites with docking score values of  − 32.76,  − 35.43, and  − 30.12 kcal/mol, respectively. More research is proposed on CS-APCR as an antibacterial and anti-SARS-COV-2 agent. SEM images of CS/PVP composite electrospun nanofibers with different ratios of CS derivatives showed smooth bead-free nanosized fibers. It was found that CS derivatives as well as CS derivatives/PVP nanofibers have higher antibacterial activity against *S. aureus* more than *E. coli*.

## Data Availability

The data will be available upon request.
